# Antioxidant resveratrol restores renal sodium transport regulation in SHR

**DOI:** 10.14814/phy2.12618

**Published:** 2015-11-24

**Authors:** Apurva A Javkhedkar, Anees A Banday

**Affiliations:** Heart and Kidney Institute, College of Pharmacy, University of HoustonHouston, Texas

**Keywords:** G proteins, hypertension, Na/K-ATPase, oxidative stress, transcription factors

## Abstract

Previously we have shown that in spontaneously hypertensive rats (SHR) renal angiotensin (Ang) II receptor (AT1R) upregulation leads to overstimulation of Na/K-ATPase by Ang II. There are reports that antioxidants can reduce oxidative stress and blood pressure (BP) in SHR, however the effect of these compounds on AT1R function remains to be determined. Therefore, we hypothesized that polyphenol antioxidant resveratrol would mitigate oxidative stress, normalize renal AT1R signaling, and reduce BP in SHR. SHR and wistar-kyoto (WKY) rats were treated with resveratrol for 8 weeks. Untreated SHR exhibited oxidative stress and enhanced renal proximal tubular Ang II-induced G-protein activation and Na/K-ATPase stimulation. Treatment of SHR with resveratrol mitigated oxidative stress, reduced BP, and normalized renal AT1R signaling. In SHR, nuclear expression of transcription factor NF-κB was increased while expression of Nrf2 was reduced. SHR also exhibited a significant decrease in renal antioxidant capacity and activities of phase II antioxidant enzymes. Resveratrol treatment of SHR abolished renal NF-κB activation, restored Nrf2-phase II antioxidant signaling and Ang II-mediated Na/K-ATPase regulation. These data show that in SHR, oxidative stress via activation of NF-κB upregulates AT1R–G-protein signaling resulting in overstimulation Na/K-ATPase which contributes to hypertension. Resveratrol, via Nrf2, activates phase II antioxidant enzymes, mitigates oxidative stress, normalizes AT1R–G-protein signaling and Na/K-ATPase regulation, and decreases BP in SHR.

## Introduction

Oxidative stress is an independent risk factor in the development of hypertension in humans and experimental animal models (Cheung and Li 2012; Khan 2012; Montezano and Touyz 2012). Although the exact mechanisms which link oxidative stress to hypertension remain to be identified, it is reported that angiotensin receptors (AT1R) play a pivotal role in the development and maintenance of hypertension during oxidative stress (Green et al. 2012; Lara et al. 2012; Putnam et al. 2012). Ang II via AT1R is a potent vasoconstrictor and modulates blood pressure (BP) by increasing vascular tone (Nguyen Dinh Cat and Touyz 2011). In addition, renal Ang II is an important regulator of sodium homeostasis and modulates sodium absorption indirectly via aldosterone release and directly by activating renal tubular sodium transporters (Banday and Lokhandwala 2008; Hattangady et al. 2012; Horiuchi et al. 2012; Bollang 2014). Previous studies from our laboratory and others have shown that oxidative stress can influence Ang II-mediated renal sodium regulation (Banday and Lokhandwala 2008). We found that oxidative stress upregulates renal AT1R which leads to an overstimulation of proximal tubular sodium transporters (Banday and Lokhandwala 2008, 2011). Also, the inhibition of sodium transporters caused by high Ang II concentration was abolished (Banday and Lokhandwala 2008, 2011).

Evidence to date indicates that antioxidant treatment ameliorates oxidative stress and reduces BP in various animal models including SHR (Welch et al. 2005; Ng et al. 2011). The mechanisms for the beneficial effects of these antioxidants are either unclear or remain confined to their ability to scavenge free radicals and thus reduce oxidative stress. However, recent evidences show that polyphenol antioxidants like resveratrol have a specific mode of action in that their antioxidant function is specifically related to their ability to modulate the activity of redox-sensitive transcription factors such as nuclear factor-E2-related Factor-2 (Nrf-2) and nuclear factor (NF)-κB (Ren et al. 2011; He et al. 2012; Rieder et al. 2012). Interestingly, it is reported that AT1R gene can be modulated by redox-sensitive transcription factors like NF-κB, SP1, and activator protein-1 (Langford et al. 1992; Cowling et al. 2002; Bogdarina et al. 2009). Therefore, the present study was designed to investigate the beneficial effect of resveratrol treatment in SHR as it relates to the effect of resveratrol on the regulation of renal transcription factors Nrf2 and NF-κB, as well as on AT1R signaling and Na/K-ATPase regulation.

## Methods

### Animals

Male SHR and WKY rats (Charles River Labs, Wilmington, MA) were divided into the following four groups: (Arinze and Kawai 2005) WKY-V, rats provided with tap water; (Asghar et al. 2006) SHR-V, rats kept on tap water; (Banday et al. 2003) WKY-R, rats provided with resveratrol in tap water; and (Banday and Lokhandwala 2011) SHR-R, rats provided with resveratrol in tap water. Resveratrol (50 mg/L) was given for 8 weeks from 3 to 11 weeks of age. All experiments were performed according to University of Houston guidelines and protocols were approved by Institutional Animal Care and Use Committee (IACUC).

### Animal surgery

Conscious BP was measured as detailed previously (Javkhedkar et al. 2012). Briefly, 5–6 weeks old rats were anesthetized with isoflurane and a midline abdominal incision was made to expose the aorta. The catheter of the radio transmitter was inserted into the abdominal aorta, guided upstream, and secured with tissue adhesive. The body of the telemetric device was placed in the abdominal cavity and sutured to the abdominal musculature. Analgesia (buprenorphine, 0.05 mg/kg SQ) was administered every 12 h for 2 days and animals were allowed to recover for 1 week prior to the recording of BP. Urinary 8-isoprostane was measured by RIA kit (Cayman, Ann Arbor, MI) and nitrotyrosine was determined by using an ELISA assay kit (Millipore, Billerica, MA). Malondialdehyde was measured by the method of Mihara and Uchiyama (Mihara and Uchiyama 1978).

### Preparation of renal proximal tubules

At the end of the resveratrol treatment rats (11-week-old) were anesthetized with sodium pentobarbital (50 mg/kg, i.p.; Sigma-Aldrich, St. Louis, MO), a midline abdominal incision was made and abdominal aorta was cannulated below the kidneys and perfused with an enzyme solution of 230 U/mL collagenase and 250 U/mL hyaluronidase (Sigma-Aldrich) as detailed previously (Marwaha et al. 2004). Proximal tubules were isolated by density-gradient centrifugation using 25% Ficoll in Krebs–Henseleit buffer B. Protein was determined by using a bicinchoninic acid kit (Pierce, Rockford, IL). Proximal tubules were homogenized and centrifuged to isolate membrane and cytosolic fractions using routine laboratory techniques (Asghar et al. 2006). Total proximal tubular antioxidant capacity was measured by using a commercially available assay kit (Cayman Chemical Company, Ann Arbor, MI). Renal proximal tubular heme oxygenase (HO-1) (assay kit from Enzo Life Sciences – Cat # ADI_EKS-800) and NAD(P):quinine oxidoreductase 1(NQO1) (rat assay kit from USCN life Sciences – Cat # E80969Ra) were measured as per the manufacturers’ protocol.

### Na/K-ATPase activity

As detailed previously (Marwaha et al. 2004), renal proximal tubules (1 mg protein) were incubated without (basal) and with Ang II (10^-10^ mol/L or 10^-6^ mol/L) at 37°C for 10 min. Following the incubation of proximal tubules with Ang II in DMEM, ^86^Rb^+^ uptake was measured for 5 min. For candesartan treatment, the tubules were preincubated with 1 *μ*mol/L candesartan prior to the addition of Ang II. The uptake was initiated by addition of 1 mL DMEM containing 1 Ci/mL ^86^Rb^+^ (Seri et al. 1988; Banday et al. 2003). Tubules were lysed with 3% sodium dodecyl sulfate (1.5 mL/well), and radioactivity was measured directly in cell lysate with a gamma counter. Protein was measured in lysate by BCA method. Na/K-ATPase activity was determined as the difference between ^86^Rb^+^ uptake in the absence and presence of ouabain (Seri et al. 1988; Banday et al. 2003).

### [^35^S]GTP*γ*S binding assay

Proximal tubular membranes (5 *μ*g protein) were incubated with 0.6 nmol/L [^35^S]GTP*γ*S and Ang II (10^−6^ mol/L) at 30°C for 60 min. Nonspecific [^35^S]GTP*γ*S binding was determined in the presence of 100 *μ*mol/L unlabeled GTP*γ*S. The reaction mixture was filtered through Millipore apparatus and radioactivity was determined by liquid scintillation counter (Beckman Coulter, Brea, CA) as described elsewhere (Marwaha et al. 2004).

### Immunoblotting and ELISA

As detailed before (Asghar et al. 2006), proximal tubular nuclear fractions were isolated using commercially available NE-PER nuclear and cytosolic protein extraction kit (Pierce). Membrane and whole cell lysate samples for immunoblotting were prepared in Laemmli buffer, resolved by SDS-PAGE, and transferred to PVDF membrane (Millipore) followed by overnight blocking with 5% bovine serum albumin (Laemmli 1970). The membranes were incubated with Gi*α* (Millipore – Cat # MAB3075) and Gq/11 (Millipore – Cat # 371754)-specific primary antibodies followed by incubation with HRP-conjugated secondary antibodies. The bands were visualized on X-ray film and quantified by Kodak Imaging System (Rochester, NY). Nuclear NF-κB and Nrf2 translocation were quantitated by commercially available rat-specific ELISA kits (Signosis, Sunnyvale CA and Mybiosource, San Diego, CA). For both Nrf2 and NK-κB ELISA assay, optical density was measured at 450 nm. The plate was also read at 540 or 570 nm and the optical density at 540 or 570 nm was subtracted from the reading recorded at 450 nm to correct the optical imperfections in the plate.

### Statistics analysis

Differences between the means were evaluated using two-way ANOVA for comparing the four groups (WKY-V, SHR-V, WKY-R, and SHR-R) and repeated measures ANOVA to analyze each group for change in BP from week 7 through 11 followed by post hoc Newman–Keuls multiple test, as appropriate. *P *<* *0.05 was considered statistically significant.

## Results

### Effect of resveratrol on BP and oxidative stress markers

Treatment of SHR and WKY rats with resveratrol for 8 weeks had no effect on body weight or food and water intake (Table1). As expected, BP was higher in SHR compared to WKY rats at 11 weeks of age (Fig.1). Treatment of SHR markedly reduced BP, however it remained significantly higher than WKY rats (Fig.1). Resveratrol had no effect on BP in WKY rats (Fig.1). SHR exhibited oxidative stress as evidenced by increased renal proximal tubular malondialdehyde and nitrotyrosine levels and urinary 8-isoprostane (Table1). These animals also had decreased proximal tubular antioxidant capacity (Table1). Resveratrol treatment normalized the levels of malondialdehyde, nitrotyrosine, and 8-isoprostane and restored antioxidant capacity in SHR (Table1). Resveratrol did not affect the oxidative milieu of WKY rats (Table1).

**Table 1:** Effect of resveratrol on body weight, food consumption, and oxidative stress markers in 11-week-old spontaneously hypertensive rats (SHR) and WKY rats

	WKY-V	SHR-V	WKY-R	SHR-R
Body weight, g	322.1 ± 7.9	312.5 ± 6.1	309.8 ± 6.1	324.1 ± 11.1
Food intake/day, g	21.04 ± 2.2	19.43 ± 2.3	18.90 ± 3.9	22.01 ± 4.01
Water intake/day, mL	43.0 ± 4.1	47.03 ± 3.1	44.22 ± 3.2	46.12 ± 5.7
Malondialdehyde, nmole/mg protein	88.1 ± 3.3	147.0 ± 6.7*	89.7 ± 6.4#	97.2 ± 4.8#
Nitrotyrosine, arbitrary units	104.0 ± 6.1	162.0 ± 7.1*	94.4 ± 4.4#	111.3 ± 7.2#
8-Isoprostane, pg/mg creatinine	55.3 ± 3.2	89.2 ± 3.9*	46.51 ± 4.2#	64.3 ± 3.9#
Antioxidant capacity, pmol/L	422.0 ± 24.1	222.6 ± 20.1*	453.1 ± 29.3#	382.2 ± 27.8#

V, vehicle (tap water), R, resveratrol.

Data (mean ± SE) for body weight and food/water intake are from *n* = 9–13 rats in each group. Data (mean ± SE) presented for nitrotyrosine, 8-isoprostane, and antioxidant capacity are from *n* = 7–9 rats in each group and the assays were performed in triplicate.

* *P *<* *0.05 versus WKY-V.

# *P *<* *0.05 versus SHR-V.

**Figure 1:**
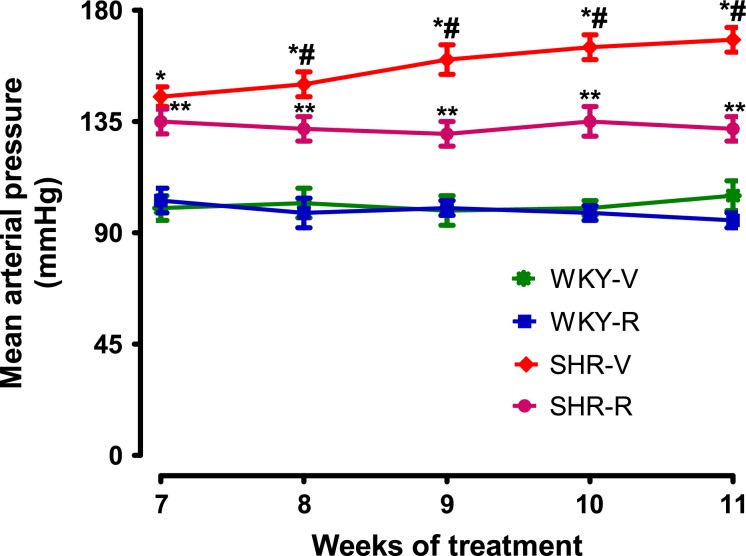
Mean arterial pressure in spontaneously hypertensive rats (SHR) and WKY. Conscious arterial pressure was continuously measured from week 7 to week 11 in all experimental groups by radiotelemetry. ^*^P <* *0.05 versus WKY-V, ^*#*^*P *<* *0.05 versus SHR-R, and ^**^P <* *0.05 versus WKY-V at respective time points, using two-way ANOVA followed by Newman–Keuls post hoc test.

### Effect of resveratrol on Nrf2 and phase II antioxidant enzyme activities

**Figure 2:**
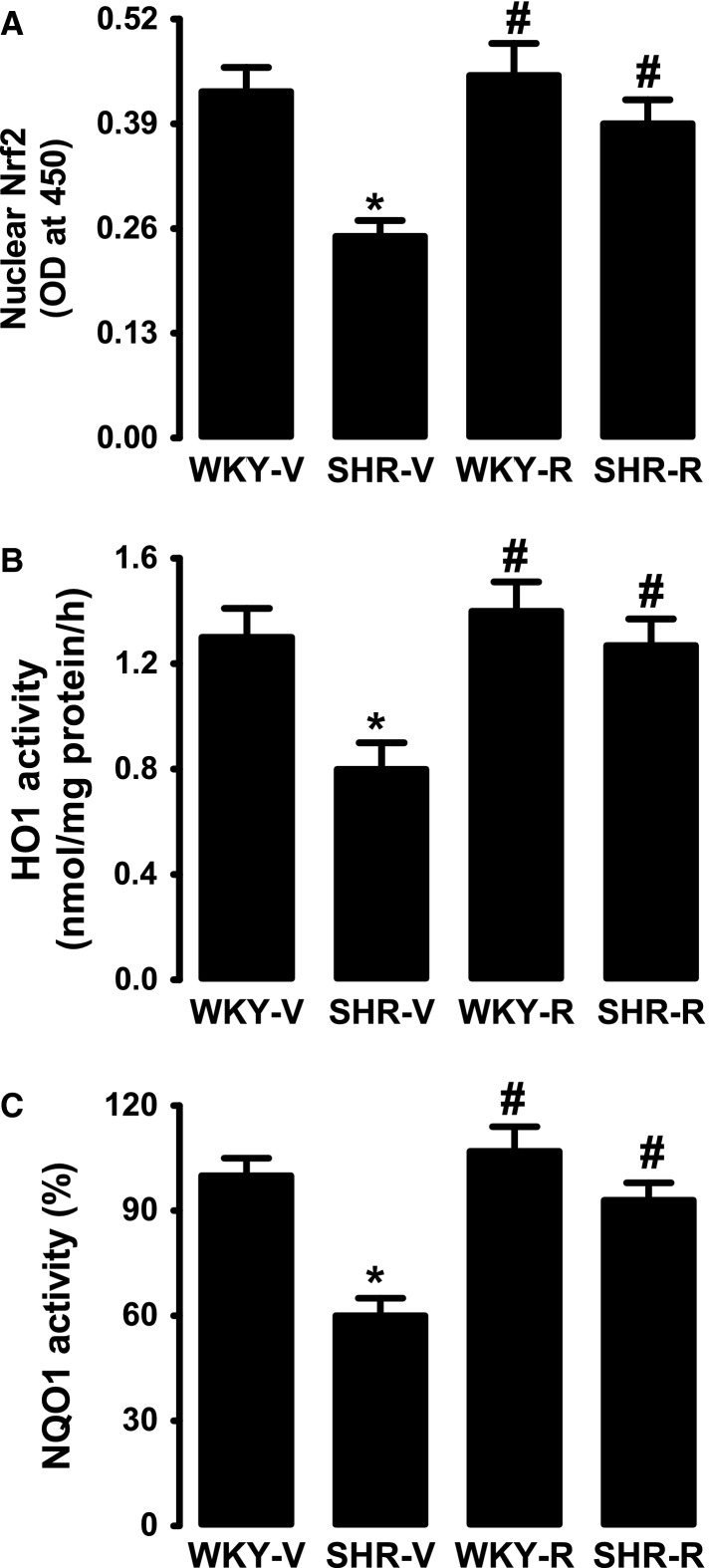
Effect of resveratrol on Nrf2 and phase II antioxidant enzymes in renal proximal tubules from WKY rats and spontaneously hypertensive rats (SHR). (A) Nrf2 nuclear expression, bars are mean ± SE, *n* = 5–7 rats in each group, and the assay was performed in triplicate. (B, C) Activities of heme oxygenase (HO-1) and NAD(P):quinine oxidoreductase 1(NQO). Data are presented as mean ± SE, *n* = 5–7 rats in each group, and the assays were performed in triplicate. ^*^P <* *0.05 versus WKY-V and ^*#*^*P *<* *0.05 versus SHR-V, using two-way ANOVA followed by Newman–Keuls post hoc test.

As shown in Figure2A, the proximal tubular nuclear Nrf2 content in SHR is markedly lower than WKY rats and resveratrol treatment restored nuclear Nrf2 in SHR (Fig.2A). Activities of phase II antioxidant enzymes HO-1 and NQO1 were significantly lower in proximal tubules of SHR compared to WKY rats (Fig.2B, C). Resveratrol treatment restored HO-1 and NQO1 activities in SHR (Fig.2B, C). Both untreated and resveratrol-treated WKY rats exhibited similar HO-1 and NQO1 activity (Fig.2B, C).

### Effect of resveratrol on Ang II-induced Na/K-ATPase activation

**Figure 3:**
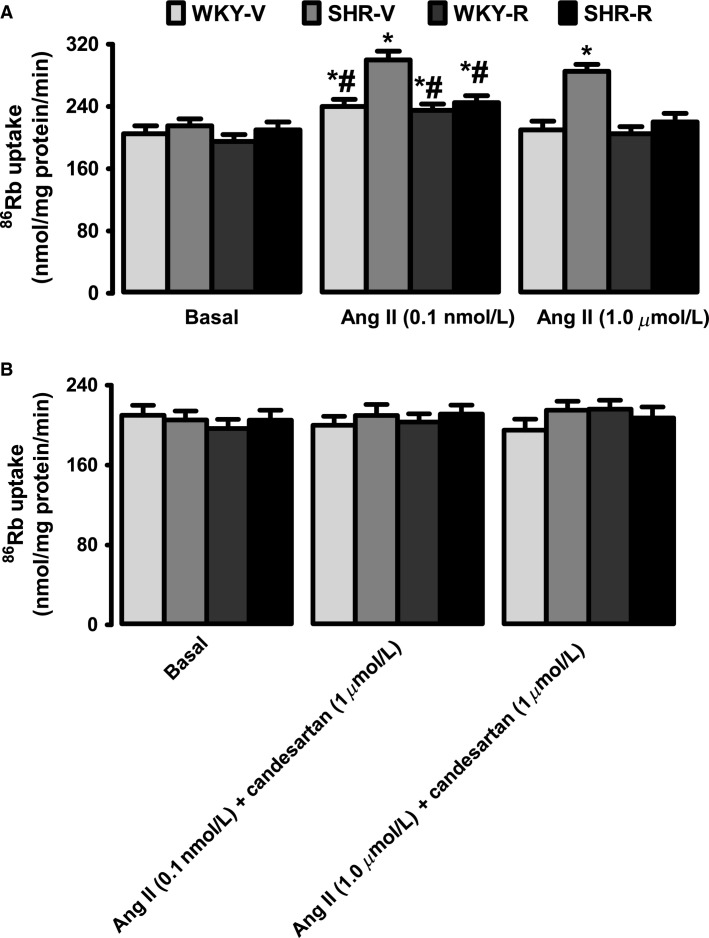
Effect of resveratrol on Ang II-induced renal proximal tubular Na/K-ATPase stimulation in WKY rats and spontaneously hypertensive rats (SHR). (A) Na/K-ATPase activity was measured as ^86^Rb^+^ uptake. Proximal tubules were incubated with low (0.1 nmol/L) or high (1 *μ*mol/L) Ang II concentrations (B) for 10 min. Proximal tubules were incubated with low or high Ang II concentrations in the presence of AT1R antagonist candesartan (Can). Data are presented as mean ± SE, *n* = 5–7 rats in each group, and the assays were performed in triplicate. ^*^P <* *0.05 versus respective basal and ^*#*^*P *<* *0.05 versus SHR-Ang II, using two-way ANOVA followed by Newman–Keuls post hoc test.

Incubation of proximal tubules with low concentration of Ang II (10^−10^ mol/L) for 10 min significantly stimulated Na/K-ATPase activity in both SHR and WKY rats (Fig.3A). However, Ang II-induced stimulation of Na/K-ATPase was markedly higher in SHR compared to WKY rats (Fig.3A). Resveratrol treatment in SHR significantly reduced the overstimulation of Na/K-ATPase in response to Ang II (Fig.3A). At a higher concentration, Ang II (10^−6^ mol/L) failed to activate Na/K-ATPase in WKY rats; yet, at this concentration Ang II continued to stimulate Na/K-ATPase in SHR (Fig.3A). Ang II-induced Na/K-ATPase stimulation decreased significantly in resveratrol-treated SHR compared to untreated SHR (Fig.3A). Resveratrol had no effect on Ang II response in WKY rats (Fig.3A). As shown in Figure3B, Na/K-ATPase activation by Ang II was sensitive to AT1R antagonist candesartan (1 *μ*mol/L) in all the groups. The basal Na/K-ATPase activity was equal in all the groups (Fig.3A, B).

### Effect of resveratrol on renal G-protein expression and AT1R–G-protein coupling

**Figure 4:**
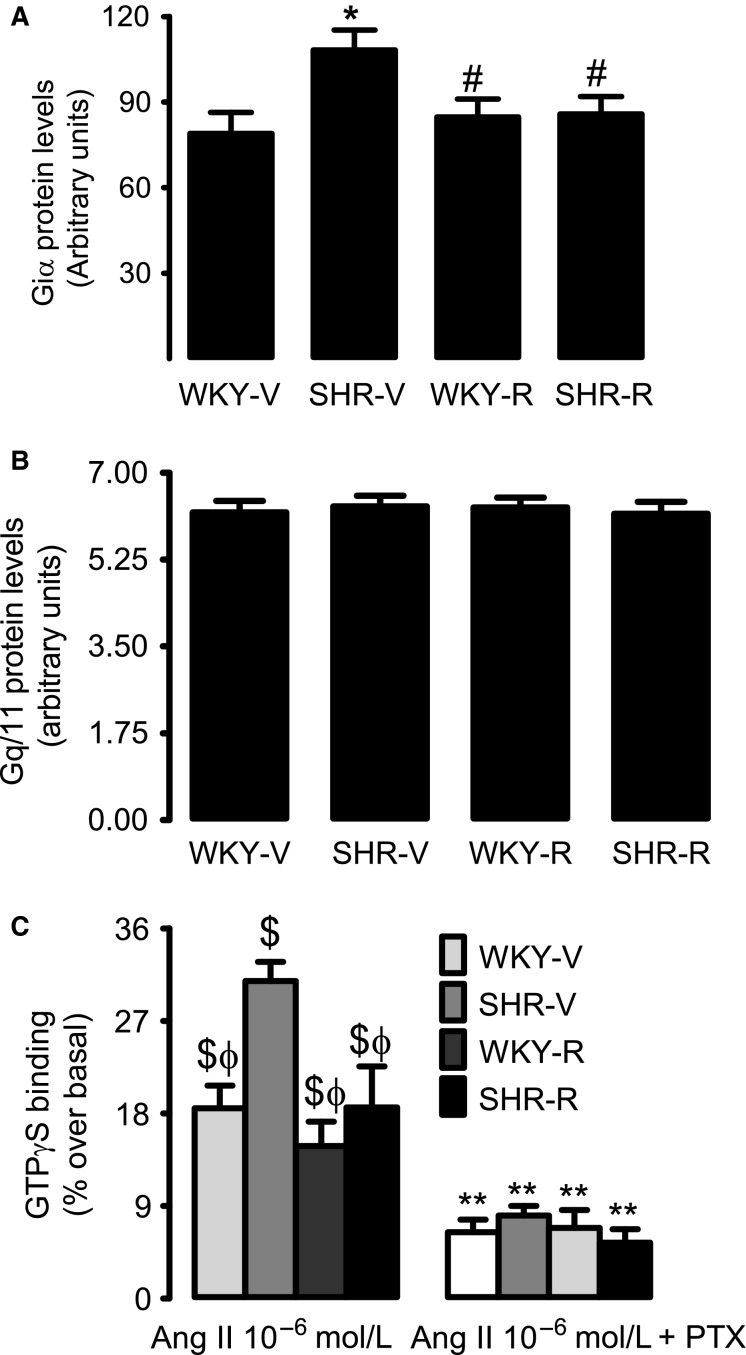
Effect of resveratrol on renal proximal tubular G-protein expression and coupling in WKY rats and spontaneously hypertensive rats (SHR). Top panels are representative western blots for (A) Gi*α* and (B) Gq/11 and bars represent mean ± SE of band densities, *n* = 6–8 rats. (C) Ang II-mediated [^35^S]GTP*γ*S membrane binding in the absence and presence of Gi*α* inhibitor pertussis toxin (PTX). Data are presented as mean ± SE, *n* = 6–8 rats in each group, and [^35^S]GTP*γ*S membrane binding was performed in triplicate. ^*^P <* *0.05 versus WKY-V, ^*#*^*P *<* *0.05 versus SHR-V, ^*$*^*P *<* *0.05 versus basal, ^*ϕ*^*P*<0.05 versus SHR-V (with Ang II 10^-6^ mol/L), and ^**^P <* *0.05 versus corresponding Ang II (10^-6^ mol/L) group, using two-way ANOVA followed by Newman–Keuls post hoc test.

Western blot analysis showed higher levels of Gi*α* protein in renal proximal tubular membranes of SHR compared to WKY rats (Fig.4A). In resveratrol-treated SHR, there was a significant decrease in Gi*α* protein expression compared to untreated SHR (Fig.4A). Resveratrol had no effect on Gi*α* expression in WKY rats (Fig.4A). Proximal tubular Gq/11 protein expression was similar in all the experimental groups (Fig.4B). Incubation of proximal tubules with 1 *μ*mol/L Ang II increased membrane [^35^S]GTP*γ*S binding in both SHR and WKY rats (Fig.4C). However, the membrane [^35^S]GTP*γ*S binding was significantly higher in SHR compared to WKY rats (Fig.4C). Resveratrol treatment normalized [^35^S]GTP*γ*S binding in SHR, but did not change [^35^S]GTP*γ*S binding in WKY rats (Fig.4C). Preincubation of proximal tubular membranes with pertussis toxin decreased [^35^S]GTP*γ*S binding in all groups (Fig.4C). However, the decrease in [^35^S]GTP*γ*S was more robust in SHR compared to WKY rats (Fig.4C), indicating that increased expression of Gi*α* could be contributing to enhanced AT1R–G-protein coupling in SHR.

### Effect of resveratrol on activation of NF-κB

**Figure 5:**
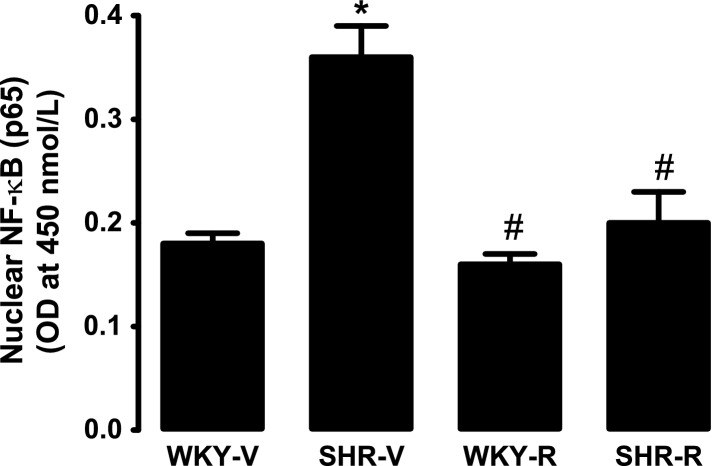
Effect of resveratrol on renal proximal tubular nuclear NF-κB (p65) expression in WKY rats and spontaneously hypertensive rats (SHR). Data are presented as mean ± SE, *n* = 5–7 rats, and the assays were performed in triplicate.^*^P <* *0.05 versus WKY-V and ^*#*^*P *<* *0.05 versus SHR-V, using two-way ANOVA followed by Newman–Keuls post hoc test.

NF-κB (p65 subunit) content was markedly higher in renal proximal tubular nuclear fraction of SHR compared to WKY rats (Fig.5). Resveratrol treatment significantly decreased nuclear p65 subunit expression in SHR while having no effect in WKY rats (Fig.5).

## Discussion

Results from our present studies show that SHRs have redox imbalance, decreased renal nuclear Nrf2 expression, and reduced activities of phase II antioxidant enzymes. SHR also exhibit increased renal NF-κB and Gi*α* protein expression and enhanced Ang II-induced G-protein coupling which leads to overstimulation of Na/K-ATPase. Resveratrol treatment of SHR restored normal redox milieu, increased Nrf2 and phase II antioxidant enzyme activities, and decreased NF-κB and Gi*α* expression. Also, resveratrol normalized Ang II-induced G-protein coupling, restored Ang II-mediated Na/K-ATPase regulation, and reduced BP.

Studies show that increase in oxidative stress contributes to the development of hypertension and antioxidant treatment decreases BP in various animal models including SHR (Welch et al. 2005; Kawakami et al. 2011; Ng et al. 2011). Consistent with these studies we also found that resveratrol treatment reduced oxidative stress and BP in SHR while having no effect in normotensive WKY rats. Our data show that SHR not only exhibit increase in oxidative stress, but the antioxidant capacity of these animals is also compromised. The exact mechanisms for this phenomenon remain unclear. However, we found a decrease in expression and activities of nuclear Nrf2 protein and cytosolic phase II antioxidant enzymes HO-1 and NQO1 indicating that Nrf2-phase II antioxidant signaling pathway is reduced in SHR. Nrf2-phase II enzyme pathway is an important redox-sensitive system which protects cells against oxidative damage (Ren et al. 2011; Copple 2012; He et al. 2012). Under normal conditions, Nrf2 is present in cytoplasm and upon receiving stimulus such as increase in oxidant levels, Nrf2 translocates to the nucleus where it increases transcription of phase II enzymes such as HO-1 and NQO1 which in turn enhance antioxidant capacity to protect cells against oxidative injury (Ren et al. 2011; Copple 2012; He et al. 2012). It was interesting to observe that despite oxidative stress, Nrf2 and phase II antioxidant signaling was reduced in SHR which could in part explain the reduced antioxidant capacity observed in these animals. We did not study the mechanism for this paradoxical downregulation of Nrf2 pathways during oxidative stress. However, we found increased expression of NF-κB in SHR and recent reports show that NF-κB interacts with Keap 1 (a Nrf2 inhibitor) which binds to cytosolic Nrf2 and leads to its proteasomal degradation and suppresses Nrf2 antioxidant enzyme pathway. It is reported that polyphenols such as resveratrol can activate Nrf2 signaling pathways and ameliorate oxidative stress (Ren et al. 2011; He et al. 2012; Reisman et al. 2012). Here, we also found that treatment of SHR with resveratrol upregulated Nrf2 and phase II enzymes HO-1 and NQO1 which contributed to the restoration of redox status in these animals. It is worth mentioning that resveratrol had no effect on BP, oxidative stress, or Nrf2-phase II enzyme signaling in WKY rats. This lack of effect is physiologically relevant because Nrf2 activation is only useful during increased oxidative stress. Taken together these data show that resveratrol activated Nrf2-phase II enzyme pathway, reduced oxidative stress, and decreased BP in SHR.

Regulation of renal proximal tubular sodium transporters by Ang II is an important mechanism for maintaining sodium homeostasis and BP (Gildea et al. 2012). We and others have shown that the regulation of renal proximal tubular Na/K-ATPase in response to Ang II is biphasic, such that picomolar–nanomolar concentrations of Ang II stimulate Na/K-ATPase, whereas at micromolar concentration, Ang II either inhibits or fails to stimulate Na/K-ATPase (Harris and Young 1977; Banday and Lokhandwala 2008). We found that in WKY rats Ang II (10^-10^ mol/L) stimulated Na/K-ATPase at low concentrations and there was no stimulation at high Ang II (10^-6^ mol/L) concentration. However in SHR, the activation of Na/K-ATPase at low concentrations of Ang II (10^-10^ mol/L) was more robust and this stimulation continued at high concentration of Ang II (10^-6^ mol/L) suggesting overstimulation of Na/K-ATPase and loss of biphasic regulation. Treatment of SHR with resveratrol normalized the Ang II-mediated Na/K-ATPase regulation indicating that oxidative stress could be a contributing factor for this phenomenon. This is further supported by the data that SHR exhibit increased AT1R–G-protein coupling which could be responsible for exaggerated Na/K-ATPase stimulation and resveratrol treatment normalized AT1R–G-protein coupling and Ang II-induced Na/K-ATPase regulation in SHR. We did not elucidate the mechanism for the loss and restoration of biphasic response as it is related to Na/K-ATPase in SHR and resveratrol-treated SHR, respectively. However, we have previously shown that a defect in NO singling is a contributor to the loss of biphasic response during oxidative stress and treatment of animals with antioxidants restores NO signaling (Banday and Lokhandwala 2008; Javkhedkar et al. 2012). Altogether, these data show that oxidative stress leads to increased signaling of AT1R causing failure of renal Ang II to regulate Na/K-ATPase, which could contribute to development of hypertension in SHR. Resveratrol, while normalizing renal AT1R signaling and Na/K-ATPase regulation, decreases BP in SHR.

To elucidate the mechanism involved in increased Ang II-mediated G-protein coupling in SHR, we determined G-protein expression in these rats. It is reported that an increase in G-protein expression per se can exaggerate AT1R signaling in obese Zucker rats (Shah and Hussain 2006). We found a significant increase in proximal tubular Gi*α* expression, while there was no change in Gq/11 protein in SHR. Furthermore, incubation of proximal tubules with pertussis toxin, a Gi*α* inhibitor, abolishes exaggerated AT1R–G-protein coupling in SHR. Treatment of SHR with resveratrol normalized Gi*α* expression and AT1R–G-protein coupling suggesting that oxidative stress could be responsible for increase in G-protein expression and coupling to AT1R in these animals.

To identify a mechanism responsible for increased Gi*α* expression, we studied the nuclear expression of NF-κB. It is well known that upon activation by oxidative stress, NF-κB is translocated to cell nucleus where it could transcriptionally upregulate gene expression (Pasparakis 2012). Our results showed higher expression of p65 subunit in nuclear fraction of proximal tubules in SHR compared to WKY rats and resveratrol treatment in SHR decreased p65 expression. These data are consistent with studies done in K562 cells that show that oxidative stress via activation of NF-κB increases Gi*α* expression (Arinze and Kawai 2005). Therefore, our results suggest that enhanced activation of NF-κB could contribute to increased Gi*α* expression leading to higher AT1R–Gi*α* protein coupling in SHR. Resveratrol while normalizing NF-κB decreases Gi*α* expression and AT1R–G-protein signaling in these rats.

### Limitation

Resveratrol treatment despite of normalizing the oxidative milieu and Na/K-ATPase regulation failed to abolish high BP in SHR. The most plausible explanation could be the fact that hypertension is a complex multifactorial disease whose etiology involves myriad of exogenous and endogenous factors. While oxidative stress could be one the contributors which was corrected by resveratrol treatment, the other factors like increased sympathetic nerve activity, reduced response to and bioavailability of nitric oxide, and defective renal dopamine system could maintain the increased BP in SHR compared to WKY rats. It is also worth noting that BP was recorded in adult animals (7–11 weeks of age) and other parameters like oxidative markers, G-protein coupling, and phase II antioxidant enzymes were measured at the end of the treatment. Therefore, no data are available to assess the effect of resveratrol on either oxidative stress or BP at earlier age. Since changes in oxidative stress or BP during young age can have a profound effect on oxidative stress, AT1R signaling, and development of BP in adult rats, further studies are needed to fully evaluate the cardiovascular benefits of polyphenols including resveratrol.

In conclusion, our results show that, in part, oxidative stress via NF-κ B activation increases AT1R–Gi*α* signaling which leads to overstimulation of Na/K-ATPase in SHR. Also, oxidative stress leads to loss of biphasic Na/K-ATPase regulation in response to Ang II. A defect in sodium regulation could contribute to increase in BP in SHR. The failure to maintain redox status in SHR could be due to a decrease in Nrf2-phase II antioxidant signaling pathway. Resveratrol treatment in SHR normalizes Nrf2-phase II enzymes pathways and mitigates oxidative stress. A reduction in oxidative stress decreases NF-κB signaling which normalizes AT1R–G-protein signaling and Ang II-mediated Na/K-ATPase regulation. These set of events contribute to a decrease in BP in resveratrol-treated SHR.

## Conflict of Interest

None declared.
